# U-251 revisited: genetic drift and phenotypic consequences of long-term cultures of glioblastoma cells

**DOI:** 10.1002/cam4.219

**Published:** 2014-05-08

**Authors:** Anja Torsvik, Daniel Stieber, Per Øyvind Enger, Anna Golebiewska, Anders Molven, Agnete Svendsen, Bengt Westermark, Simone P Niclou, Thale Kristin Olsen, Martha Chekenya Enger, Rolf Bjerkvig

**Affiliations:** 1 Department of Biomedicine, University of BergenBergen, Norway; 2 NorLux Neuro-Oncology Laboratory, Department of Oncology, Centre de Recherche Public de la Santé (CRP-Santé)Luxembourg, Luxembourg; 3 Department of Neurosurgery, Haukeland University HospitalBergen, Norway; 4 The Gade Institute/Section of Pathology, Institute of Medicine, University of BergenBergen, Norway; 5 Department of Pathology, Haukeland University HospitalBergen, Norway; 6 Department of Genetics and Pathology, Uppsala UniversityUppsala, Sweden; 7 Section for Cancer Cytogenetics, Oslo University Hospital, The Norwegian Radium HospitalOslo, Norway

**Keywords:** aCGH, cell lines, cross-contamination, GBM, in vitro models, STR, U251, U373

## Abstract

It is well known that in vitro subculture represents a selection pressure on cell lines, and over time this may result in a genetic drift in the cancer cells. In addition, long-term cultures harbor the risk of cross-contamination with other cell lines. The consequences may have major impact on experimental results obtained in various laboratories, where the cell lines no longer reflect the original tumors that they are supposed to represent. Much neglected in the scientific community is a close monitoring of cell cultures by regular phenotypic and genetic characterization. In this report, we present a thorough characterization of the commonly used glioblastoma (GBM) model U-251, which in numerous publications has been wrongly identified as U-373, due to an earlier cross-contamination. In this work, the original U-251 and three subclones of U-251, commonly referred to as U-251 or U-373, were analyzed with regard to their DNA profile, morphology, phenotypic expression, and growth pattern. By array comparative genomic hybridization (aCGH), we show that only the original low-passaged U-251 cells, established in the 1960s, maintain a DNA copy number resembling a typical GBM profile, whereas all long-term subclones lost the typical GBM profile. Also the long-term passaged subclones displayed variations in phenotypic marker expression and showed an increased growth rate in vitro and a more aggressive growth in vivo. Taken together, the variations in genotype and phenotype as well as differences in growth characteristics may explain different results reported in various laboratories related to the U-251 cell line.

## Introduction

Since the isolation of the first human immortal cancer cell line (HeLa; from a cervical cancer) in 1952, cell lines represent important biological model systems that are both easily accessible and easy to handle. Cell lines have thus facilitated cancer research in many ways, such as the study of functional aspects of tumor transformation and gene expression, the elucidation of mechanisms of resistance to therapy, drug screening, and the development of new therapeutic principles. Cancer cell lines also represent important tools to study genetic aberrations and molecular pathways in cancer. Cell lines are known to provide more consistent and reproducible research data than the more heterogeneous and more limited biopsy material, however, subculture may select for a specific genetic clone within the patient biopsy. Cell lines thus represent only a small subpopulation of the original tumor. Furthermore, long-term tissue cultures are susceptible to genetic drift, which may result in altered phenotypes and growth patterns. Also the consistency and reproducibility of cell line studies between different laboratories represents a major challenge based on genetic drift upon long-term subculture and potential cross-contamination.

Glioblastoma (GBM) represents a tumor of complex cellular heterogeneity, and numerous GBM cell lines have been established as tools to unravel molecular mechanisms related to the disease. In this study, we have assessed in detail the genetic drift in the commonly used U-251 GBM cell line, which is widely used as a GBM model. The U-251 cell line was established at the Wallenberg laboratory, Uppsala, Sweden, almost more than 40 years ago along with several other cell lines derived from human gliomas 1–3, and was derived from a male patient with malignant astrocytoma 2. In addition to the original U-251, two subclones with different morphology have been described for U-251, namely the astrocytoid U-251MGAg Cl1 and the fascicular U-251MGsp 4.

In 1999, it was reported that another GBM cell line (U-373) was cross-contaminated by U-251 5, and it was later shown that the misidentified U-251 cells had been wrongly distributed by all the major cell banks 6,7, (http://www.lgcstandards-atcc.org/MisidentifiedCellLines/tabid/1171/Default.aspx, http://www.hpa.org.uk). According to a comprehensive list of misidentified cell lines published by Capes-Davis and colleagues 8, the U-251 cell line has also cross-contaminated the so called human gliosarcoma cell line KNS-89 and the human GBM cell lines SNB-19 and TK-1 (http://www.cellbankaustralia.com/Misidentified-Cell-Line-List/default.aspx). Despite repeated efforts to clear this confusion, researchers around the world have continued to use the misidentified U-251 along with the original U-251. A Pubmed search for U-373 returns 731 results (September 2013), however, by a review of these articles it is clear that it is rarely clarified if the authors have used the true U-373 or the cross-contaminated U-251 cell line. This is also the case for the most recently published articles, in spite of a considerable focus on cell line misidentification that has been extensively addressed over the past years 8–11. In the summer of 2010, the original U-373 line was made available at the Health Protection Agency (HPA) cultures in the UK, and the short tandem repeat (STR) profile of this cell line clearly differs from that of the U-251 cell line and the misidentified U-251, wrongly distributed as “U-373” (http://www.Hpa.org.uk).

The objective of this study was to characterize the various subclones of U-251 and compare them to the original U-251MG obtained from Uppsala, Sweden. When working with these various clones in our laboratory, we noticed several differences in their cellular morphology and behavior, which led us to perform a functional comparison with regard to cell growth in vitro and in vivo, clonogenicity, and radiation resistance. To determine the genetic profiles of the cell lines we performed high-resolution array comparative genomic hybridization (aCGH) profiling. The aCGH data showed a typical cell line profile for the long-term passaged subclones, with accumulation of additional genetic changes compared to the original U-251MG (collected from cells frozen in Uppsala in 1969), which had a profile more consistent with human GBMs with several focal chromosomal amplifications and deletions. These data demonstrate how aCGH can be used to evaluate if subclones carry cell line-specific DNA copy number alterations. Our data show that subclones derived from U-251MG have experienced extensive genetic alterations indicative of genetic drift. This genetic drift is accompanied by phenotypic changes such as altered morphology, variable cell surface marker expression, increased growth rate and more aggressive cell growth both in vitro and in vivo.

## Materials and Methods

### Cell lines and cell culture

U-251N was kindly provided by Dr. J. Carlsson, Uppsala University, Sweden. U-251-FGA20gain is a subclone of U-251N. U-251-4q12 was obtained from ATCC as “U-373” HTB-17. U-251MG was stored at the Uppsala University, Sweden, at passage 64 in 1969 and thawed in November 2011. U-251MG was cultured in minimum essential medium (MEM) (Lonza, Basel, Switzerland) supplemented with 10% fetal bovine serum (FBS), 4mmol/L l-Glutamine, 100 U/mL penicillin, and 100 *μ*g/mL streptomycin. In addition, U-251MG cells were also cultured in the medium which was routinely used for the other subclones, namely Dulbecco's modified Eagle's medium (DMEM; Sigma-Aldrich Inc., St. Louis, MO) supplemented with 10% FBS, 3.2% nonessential amino acids (NEEA), 4 mmol/L l-Glutamine and 100 U/mL penicillin, 100 *μ*g/mL streptomycin. All supplements were from Lonza. The cells were cultured at 37°C and 5% CO_2_.

### Small tandem repeat analysis

Genomic DNA was purified by the DNeasy Blood & Tissue Kit (Qiagen, Hilden, Germany) according to the manufacturer's protocol for purification of total DNA from animal cells. The AmpFlSTR Profiler Plus PCR Amplification Kit (Applied Biosystems, Foster City, CA) was used according to the manufacturer's protocol. This kit amplifies nine tetranucleotide STR loci and the amelogenin (sex determination) locus in a single reaction. The samples were run and allele sizes interpreted on an ABI3100 Genetic Analyzer (Applied Biosystems). We also confirmed that none of our cells matched the STR profile of the true U-373 cell line (kindly provided by HPA cultures, (http://www.HPA.org.uk).

### aCGH analysis

Genomic DNA was extracted from cell lines using the DNAeasy Blood and Tissue Kit (Qiagen) following the manufacturer's instructions. DNA was eluted in water, fragmented to an average size of 200–500 bp using DNAse1 (rDNAse1, Ambion, Life Technology Ltd., Paisley, UK) and labeled using the BioPrime aCGH Genomic labeling Kit (Invitrogen, Paisley, UK) and Cy3 and Cy5 dyes purchased from GE Healthcare (Chalfont St. Giles, UK) following standard protocols for Agilent aCGH. Commercially available female DNA pooled from multiple anonymous donors (Promega, Madison, WI) was used as a reference for each of the aCGH experiments. Labeled DNA was competitively hybridized to SurePrint G3 Human 2 × 400 k CGH microarrays (Agilent Technologies, Santa Clara, CA) following standard Agilent protocols. The slides were scanned at 3 *μ*m resolution using the Agilent High-Resolution Microarray scanner and the image data were extracted using Feature Extraction (Agilent Technologies). FE extraction files were imported into Genomic Workbench 7.0 (Agilent Technologies) for visualization and analysis. Aberrations were called using the Aberration Detection Method 2 (ADM2) algorithm with a threshold setting of 25, centralization on with threshold of 25, and an aberration filter min Probes = 3 and minAvgAbsLogRatio = 0.25. The ADM-2 algorithm identifies all aberrant intervals in a given sample with consistently high or low log ratios based on the statistical score that represents the deviation of the average of the log_2_ ratios from the expected value of zero. Hierarchical clustering was done by using the clustering analysis implemented in the Genomic Workbench software. This is based on Euclidian distance calculations using aberration scores.

### Gene expression analysis

Total RNA was isolated from cultured cells using Trizol reagent (Life Technologies Ltd., Paisley, UK). cDNA was synthesized by using iScript cDNA Synthesis Kit (Bio-Rad Laboratories Inc., Hercules, CA). Amplification and quantification of the platelet-derived growth factor receptor *α* (PDGFR*α*) gene was done by quantitative PCR (qPCR) using the following primer sequences (5′→3′): Forward primer: GGCCGTGGGCACGCTCTTTA; Reverse primer: ACCAGGAACGCCGGATGGGA. As internal control we used 18S ribosomal RNA with the following primer sequences (5′→3′): Forward primer: CGGCTACCACATCCAAGGAA; Reverse primer: GCTGGAATTACCGCGGCT. Real-time fluorescence detection was performed by using iQ SYBR Green Supermix (Bio-Rad Laboratories Inc.), and the samples were run on a Roche Light Cycler 480 (F. Hoffmann-La Roche Ltd, Basel, Switzerland). The following temperature settings were used: Initial denaturation at 95°C for 5 min, followed by 35 cycles of denaturation at 95°C for 20 sec, annealing at 60°C for 20 sec, and extension at 72°C for 20 sec. Melting curves for the amplicons were performed after the last PCR-cycle with a temperature interval from 55°C to 95°C. Fold change was calculated by subtracting the cycle threshold (Ct) value of the reference sample from the target sample to obtain a ΔCt value. When comparing two samples, ΔΔCt is the difference between the two samples and the fold change = 2^ΔΔCt^. Fold change for each U-251 subclone was calculated relative to U-251MG.

### DNA ploidy analysis and cell cycle distribution

Subconfluent monolayers were harvested by trypsinization, washed, and fixed in ice-cold 70% ethanol, added dropwise while vortexing. The samples were stored at 4°C until use. Prior to analysis, the samples were washed in phosphate buffered saline (PBS; Sigma-Aldrich), resuspended in 1 mg/mL RNase (Sigma-Aldrich) and 50 *μ*g/*μ*L propidium iodide (PI; Sigma-Aldrich), and incubated for 30 min at room temperature prior to analysis on a FACS Accuri C6 (Accuri Cytometers Inc., Ann Arbor, MI). Lymphocytes were used as an internal diploid control and prepared for DNA analysis as described above. Cell cycle data were analyzed in ModFit LT 3.3 (Verity Software House, Topsham, ME). DNA Index (DI), which measures variations in ploidy, was calculated by the following formula: Mean G1 DNA fluorescence of cell sample/mean G1 DNA fluorescence of lymphocytes. Aneuploid cells are defined as cells which differ with more than 10% from a diploid DI = 1.0.

### Giemsa banding and karyotyping

Cell cultures were treated with colcemid (Gibco by Life Technologies, Grand Island, NY) and harvested manually according to Mandahl et al. 12. The chromosomal preparations were then G-banded using Wright stain (Merck KGaA, Darmstadt, Germany). In order to describe ploidy, the number of chromosomes in each metaphase was counted manually.

### Immunocytochemistry

Cells seeded for immunocytochemistry were fixed and permeabilized in cold methanol for 10 min at −20°C, followed by cold acetone for 1 min at −20°C. The cells were incubated in 1× PBS with 0.5% bovine serum albumin (BSA; Sigma-Aldrich) for 15 min, prior to antibody staining with mouse anti-*α*-Tubulin IgG1 MAb (Sigma-Aldrich) for 45 min at 37°C. After washing twice in 1× PBS the secondary antibody FITC-conjugated goat-anti-mouse IgG1 antibody (Southern Biotech, Birmingham, AL) was applied for 45 min at 37°C. The cells were then washed and mounted with Vectashield with DAPI (Vector Laboratories Inc., Burlingame, CA). Imaging was performed on a Leica SP2 confocal microscope.

### Cell surface marker phenotyping

A single-cell suspension of cultured cell lines was obtained by trypsinization (0.25% trypsin, Lonza) at 37°C for 2–3 min. For cell membrane staining cells were resuspended in Hank's balanced salt solution w/o Ca^2+^/Mg^2+^ (HBSS), 2% FBS, 10 mmol/L HEPES pH 7.4 buffer (10^6^cells/100 *μ*L/test) and incubated with LIVE/DEAD® Fixable Dead Cell Stains (Invitrogen, Life Technology Ltd.; 1 *μ*g/mL) and appropriate pre-conjugated antibodies (CD133/1-PE, Miltenyi Biotec, Bergisch Gladback, Germany, 10 *μ*L/test; CD15-PE Immunotools, Friesoythe, Germany, 10 *μ*L/test; A2B5-APC Miltenyi Biotec, 10 *μ*L/test; CD44-FITC, Immunotools, 10 *μ*L/test) 30 min in the dark. Data acquisition was performed with a FACS Aria™ SORP cytometer (BD Biosciences, San Jose, CA) and flow graphs were prepared with the FlowJo software (Tree Star, Inc., Ashland, OR).

### Cell proliferation

Growth curves were generated by plating 7000 cells/cm^2^ of each subclone was plated in duplicate in T25 tissue culture flasks. Every 24 h, two parallel flasks were trypsinized and counted. The experiment was repeated four times. Population doubling level (PDL), that is the total number of times a cell line doubles its population during a given time period in vitro, was calculated using the following formula: PDL = (log_10_*F* − log_10_*I*)/log (2), where *F* indicates the number of cells at the end of the passage and *I* equals the number of cells initially plated. Population doubling time (PD) was calculated for a selected interval during the logarithmic growth phase, by the formula: hours in culture/PDL.

The fraction of actively proliferating cells was measured by BrdU incorporation, using the FITC BrdU Flow Kit (BD Biosciences). Samples were prepared according to the manufacturer's instructions and analyzed on a FACS Accuri C6 (Accuri Cytometers Inc.). Cell cycle distribution in G1/G0, S, and G2M phases were analyzed by the FlowJo software.

### Clonogenic assays and irradiation

Clonogenic assays were performed as described previously 13. In short 200–375 cells/well were plated in six-well plates in triplicates, and cultured in conditioned media at 37°C, 5% CO_2_ for 10 days (>6 PD). After incubation, the cells were fixed in fixation-staining-solution consisting of 6% v/v glutaraldehyde and 0.5% w/v crystal violet (both reagents from Sigma-Aldrich) in H_2_O. A colony was defined as a cluster of minimum 50 cells and plating efficiency (PE) was calculated as described 13. PE is the ratio of the number of colonies to the number of cells seeded. *P*-values were calculated by GraphPad Software (www.graphpad.com).

In vitro doses of ionizing radiation were 2, 5, and 10 Gy, and were given at a rate of 3.1 Gy/min with a Clinac 600C/D linear accelerator irradiator (Varian Medical Systems Inc., Palo Alto, CA) to cells plated for clonogenic growth and allowed to adhere prior to irradiation. Surviving fraction was calculated as described 13 and is based on the number of clones surviving irradiation, correlated to the PE of nonirradiated control cells.

### In vivo experiments

Immunodeficient NOD.CB17.Prkdc^scid^NOD/SCID mice (Jackson Laboratory, Bar Harbor, ME) were anesthetized with Hypnorm-Dormicum (0.4 mL/kg) s.c., whereupon the head was secured in a stereotactic frame (Benchmark; Neurolab, St Louis, MO) and a short longitudinal incision was made in the scalp exposing the calvarium. A burr hole was made 0.5 mm posterior to the bregma and 1.5 mm to the right of the sagittal suture using a micro-drill with a bit diameter of 2.9 mm. A Hamilton syringe was introduced to a depth of 1.5 mm below the brain surface, and 5 × 10^4^ tumor cells were slowly injected into the brain. Each U-251 subclone was implanted in five animals. The mice were monitored on a daily basis, and were sacrificed upon signs of tumor burden/sickness, such as weight loss, stooped posture, and altered behavior. The experiments were approved by the Norwegian Animal Research Authority. Kaplan-Meier survival curves were generated using the GraphPad Software.

## Results

### STR analysis confirms a common origin of all U251 subclones

We analyzed various batches of U-251 by STR fingerprinting, and detected that we had three subclones of the U-251 cell line: U-251N which is quite similar to the STR profile published for U-251 7,14; one clone which was quite similar to U-251N but with a gain at FGA 20, resulting in three alleles at this locus (hereafter named U-251-FGA20gain); and U-251-4q12 (formerly misidentified as U-373) which is more heterogeneous at each locus than the other two (Table1). The gain at FGA 20 in U-251-FGA20gain is most likely a result of genetic drift during subculture. FGA is considered a mutational active locus 15. Multiple alleles may also represent gene duplication events; however, the aCGH analysis could not detect any duplication at this site in this sample (data not shown).

**Table 1:** STR profile of U-251.

Cell name	STR designation																				
	AMEL		D8S1179		D21S11		D18S51		D3S1358		vWA		FGA			D5S818		D13S317		D7S820	
U-251MG	X	Y	13	15	29	30	13	15	16	17	16	18		21	25	11	12	10	11	10	12
U-251-4q12	X	Y	13	15	29	30	13		16	17	16	18		21	25	11	12	10	11	10	12
U-251N	X		13	15	29		13		16	17	16	18		21	25	11		10	11	10	12
U-251-FGA20gain	X		13	15	29		13		16	17	16	18	20	21	25	11		10	11	10	12

Allele pattern of U-251 clones as analyzed by STR.

When comparing the STR profiles of these subclones to the original U-251MG, obtained from Uppsala University, Sweden, at passage 64, we could confirm that these cells have the same origin (Table1). Loss of heterozygosity (LOH) at one or more STR alleles is frequently found in cancer tissues and cancer cell lines 16,17, however, the STR profile of the original low-passage U-251MG show complete allelic heterogeneity, indicating that it has undergone less selection pressure and genetic drift in culture.

### aCGH profiles reveal closer relation of low-passaged U-251MG to human GBMs compared to long-term passaged subclones

**Figure 1:**
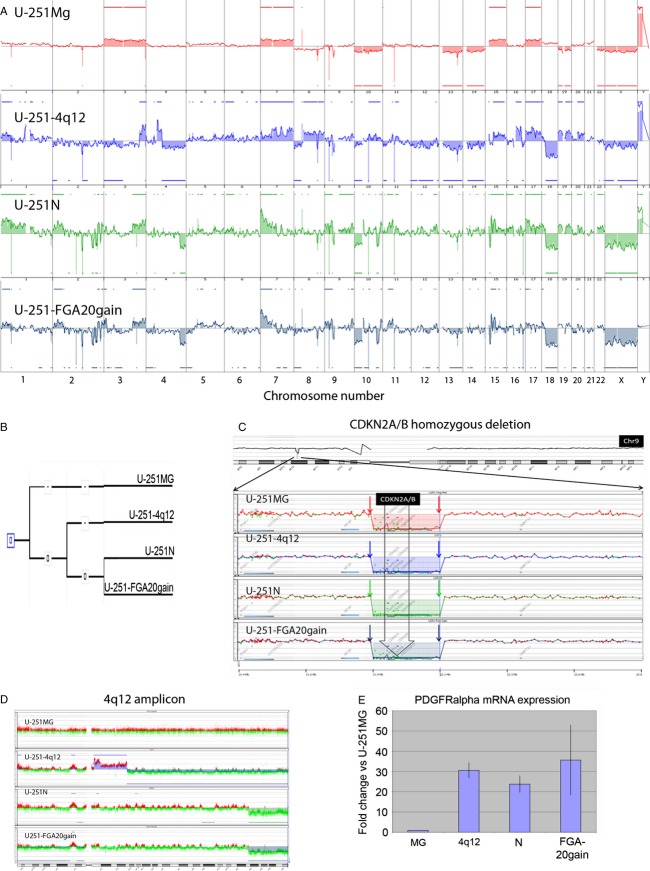
Array comparative genomic hybridization (CGH) data of U-251 clones reveal accumulation of genetic alterations after long-term culture. (A) The aCGH profile shows complete chromosomal duplications and hemizygous losses (trisomies and monosomies) in U-251MG typical of primary glioblastoma (GBM), while the profiles of the other subclones have a more complex pattern. Several homozygous deletions (arrows) are detected (B) Eucledian distance calculation indicates that U-251-4q12 diverged earlier from an ancestral U-251MG clone than U-251N and U-251-FGA20gain, and that the divergence of U-251 and U-251-FGA20gain is a relatively late event. (C) All subclones show homozygous deletion of CDKN2A (p16/p14 ARF). (D) U-251-4q12 has an amplification at chromosome 4q12. (E) platelet-derived growth factor receptor *α* (PDGFR*α*) mRNA expression is increased in all three long-term passaged U-251 subclones compared to U-251MG.

We used aCGH to determine the genetic profile of the U-251 variants. The aberration profile of U-251MG represented a typical GBM chromosome profile and differed from the other subclones, whereas the overall aberration profile between U-251-4q12, U-251N, and U-251-FGA20gain was quite similar (Fig.1A). U-251MG showed an amplification of chromosomes 3, 7, 15, and 17, as well as a deletion of chromosomes 10, 13, and 14. Interestingly, the long-term cultured subclones U-251-4q12, U-251N, and U-251-FGA20gain all showed loss on the 18q11-23 locus, which is commonly deleted in cell cultures 18. The number of aberrant intervals (gains or losses) was 27 in the U-251MG clone and 115, 160, and 138 in the U-251-4q12, U-251N and U-251-FGA20gain subclones, respectively, indicative of a high degree of genetic drift in these long-term cell cultures.

We further established that the U-251N and U-251-FGA20gain cell lines clustered together compared to U-251MG and U-251-4q12 (Fig.1B). This indicates that U-251-4q12 diverged early from the ancestral U-251MG clone and that the divergence of U-251-FGA20gain from U-251N was a relatively late event. Samples U-251MG, U-251-4q12, and U-251N were cytogenetically male as they all carry the male Y chromosome as determined by aCGH whereas U-251-FGA20gain has lost the Y chromosome (Fig.1A). Interestingly, the male amelogenin marker was not recognized in the U-251N sample when analyzed by STR profiling (Table1), indicating loss of the amelogenin locus but not the whole Y chromosome in this sample.

A tool for unambiguously tracing common lineages in different cell clones is to focus on homozygous deletions and their chromosomal breakpoints. If these are shared between several cell lines it is evident that these cell lines have a common ancestor. We have identified and mapped several common homozygous deletions in all four cell lines studied. For example CDKN2A (p16/p14 ARF) homozyguous deletion was identified in all four cell lines, and the chromosomal breakpoints were identical supporting the monoclonal origin of the four cell lines (Fig.1C). Furthermore, the accumulation of additional homozygous deletions over time can also be used to trace the lineages of the different subclones (see examples in Table2).

**Table 2:** Homozygous deletions in U-251 subclones.

Cell line	Chr	Cytoband	Start (bp on Chr)	Stop (bp on Chr)	# Probes	Deletion (mean log2 ratio)	*P*-value	Gene names
U-251MG	Chr9	P21.3	21908956	22148414	37	−2,598,488	0.000	C9orf53, CDKN2A, CDKN2BAS, CDKN2B
	Chr11	q11	55124730	55187341	12	−2,848,753	1.553e-161	OR4C11, OR4P4, OR4S2
U-251-4q12	Chr2	q22.1	141427652	142052932	119	−310,064	0.000	LRP1B
	Chr9	p21.3	21908956	22148414	37	−3,344,793	0.000	C9orf53, CDKN2A, CDKN2BAS, CDKN2B
	Chr10	q21.3	67748408	67784515	9	−4,727,192	5.978e-160	CTNNA3
	Chr11	q11	55124730	55187341	12	−4,244,455	7.253e-209	OR4C11, OR4P4, OR4S2
U-251N	Chr1	p33	51135519	51218298	18	−3,424,784	1.032e-151	FAF1, CDKN2C
	Chr2	q13	112607949	112672009	14	−3,144,469	8.919e-220	FBLN7
	Chr2	q22.1	14127652	142052932	119	−5,079,372	0.000	LRP1B
	Chr9	p21.3	21908956	22148414	37	−386,988	0.000	C9orf53, CDKN2A, CDKN2BAS, CDKN2B
	Chr10	q21.3	67748408	67784515	9	−434,557	2.586e-149	CTNNA3
	Chr11	q11	55124730	55187341	12	−4,281,094	4.431e-205	OR4C11, OR4P4, OR4S2
	Chr16	q23.1	77669056	77772300	22	−4,400,907	0.000	WWOX
U-251-FGA20gain	Chr2	q13	112607949	112672009	14	−299,373	6774e-186	FBLN7
	Chr2	q22.1	14127652	142052932	119	−440,903	0.000	LRP1B
	Chr9	p21.3	21908956	22148414	37	−3,257,158	0.000	C9orf53, CDKN2A, CDKN2BAS, CDKN2B
	Chr10	q21.3	67748408	67784515	9	−4,369,498	2.843e-140	CTNNA3
	Chr11	q11	55124730	55187341	12	−3,963,717	2.537ee-209	OR4C11, OR4P4, OR4S2
	Chr16	q23.1	77669056	77772300	22	−3,828,185	0.000	WWOX

White background, deletions common to all; black background, deletions common to 3 subclones; light gray background, common to U-251N and U-251-FGA20gain; dark gray background, proprietary to U-251N.

Unlike many primary GBM samples, we did not detect typical focal gene amplification events in the U-251N or U-251-FGA20gain cell lines. This is not surprising as such extra-chromosomal elements are typically lost in cell culture. For instance, GBMs frequently carry amplification of the endothelial growth factor receptor (EGFR) gene at chromosome 7. Chromosome 7 is duplicated in the U-251MG cell line, and the descendants all have a duplication of the EGFR locus, albeit the overall pattern of chromosome 7 of the descendant cell lines is more complex. The locus is thus duplicated in the U-251 subclones; however, the cell lines do not have a focal amplification of this region (data not shown).

In the U-251-4q12 cell line a complex amplified structure was detected on chromosome 4 including PDGFR*α*, receptor tyrosine kinases KIT, and vascular EGFR 2 (VEGFR2) (Fig.1D). In order to determine if the 4q12 amplicon was functional with respect to PDGFR*α* expression, we performed qPCR to determine the expression level of the PDGFR*α* mRNA. Interestingly, all long-term passaged subclones showed a similar upregulated expression level of PDGFR*α*, which differed significantly from the PDGFR*α* expression in U-251MG (*P* ≤ 0.025, Fig.1E), therefore, it appears that subculturing selects for high PDGFR*α* expression independent of 4q12 amplification in this cell line.

### Variations in DNA ploidy and karyotype

**Figure 2:**
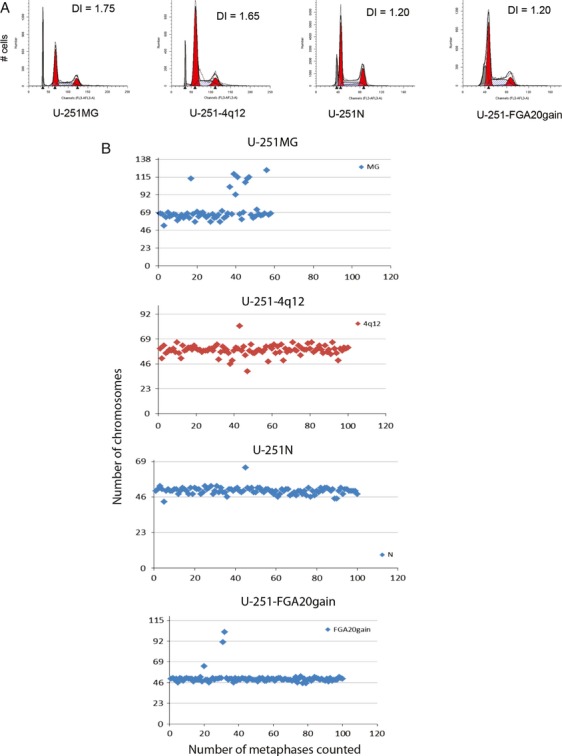
DNA ploidy and karyotyping. Flow cytometric DNA ploidy analyses show that the U-251 subclones differ in their DNA ploidy. Lymphocytes (representing diploid DNA) are shown in gray. (A) Manual count of chromosomes in G-banded metaphases showing different ploidy levels in the U-251 subclones.

DNA ploidy analysis shows that the four subclones also vary in their DI, and interestingly, the original U-251MG and U-251-4q12 are more aneuploid than the other two long-term passaged clones. U-251MG and U-251-4q12 have DI of 1.75 ± 0.07 and 1.65 ± 0.08, respectively, while U-251N and U-251-FGA20gain have DI of 1.20 ± 0.03 and 1.20 ± 0.04, respectively (Fig.2A). This variation in DNA ploidy was further confirmed by karyotyping which showed a median chromosome number of 66 for U-251MG, 59 for U-251-4q12, and 50 for both U-251N and U-251-FGA20gain (Fig.2B).

### The U.251 subclones show alterations in cellular morphology, growth pattern, and cell surface marker expression

**Figure 3:**
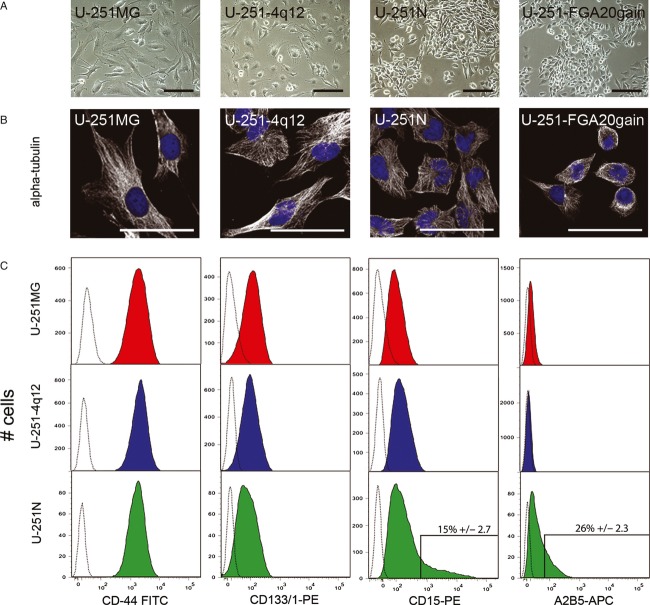
Phenotypic characterization and DNA analysis of U-251 clones. (A) Phase contrast images of U-251MG, U-251-4q12, U-251N, and U251-FGA20gain show variation in morphology and growth pattern. Scale bars = 100 *μ*m. (B) *α*-tubulin staining of U-251 subclones show variation in size and cytoskeleton organization. Scale bars = 50 *μ*m. (C) Flow cytometric histograms of cell surface marker expression profiling of U-251MG, U-251-4q12, and U251N shows high level of CD44 expression and low levels of CD133/1 and CD15. Subpopulations of CD15^high^ and A2B5^high^ cells are present only in U-251N cells (boxed area). Expression levels of analyzed markers are presented in comparison to negative controls (“black dotted” profiles).

U-251MG and U-251-4q12 cells are quite similar with respect to morphology and growth pattern, but they clearly differ from that of U-251N and U-251-FGA20gain cells (Fig.3A). U-251MG and U-251-4q12 grow evenly distributed within the culture flasks while U-251N and U-251-FGA20gain grow in clusters. Also the morphology of the cells was different. Cytoskeleton staining with *α*-Tubulin shows a more compact cytoskeleton with fewer protrusions in the longest passaged subclones compared to U-251MG. Also the cell size decrease with increased subculture (Fig.3B, all scale bars = 50 *μ*m). Flow cytometric analysis of cell membrane markers revealed that U-251MG, U-251-4q12 and U-251N cells express a uniform high level of classical glioma cell markers such as CD44 (Fig.3C), CD56, CD90, and CD29 (not shown). All cell lines also expressed low levels of CD133/1 and CD15, which were previously associated with glioma cancer stem-like cells (CSCs); however, only U-251N cells displayed a CD15^high^ subpopulation (15 ± 2.7%). A2B5 was detected at a very low level in U-251MG and U-251N cells, where again A2B5^high^ subpopulation (26 ± 2.3%) was detected only in the latter. As CD15^high^ and A2B5^high^ cells are only present in U-251N cells and not in the original U-251MG, indicates an acquisition of these markers during culture.

### Cell lines experience increased cell growth and clonogenicity upon in vitro passaging

To compare the proliferation rate between the four subclones, we performed growth curve analyses, determined the PD time, and the proportion actively cycling cells by BrdU analysis. The growth rate of U-251MG was measured in both MEM-based medium and DMEM-based medium. After an initial boost in the growth factors rich DMEM-based medium, the growth rate of U-251MG was similar in both culture media. U-251MG and U-251-4q12 grow more slowly than the other two (Fig.2A), with a PD of 33.05 ± 5.76 and 33.63 ± 3.28 h, respectively, whereas the PD for U-251N is 23.79 ± 2.26 h and that of U-251-FGA20gain is 21.09 ± 1.23 h (average from four growth curve replicates). The BrdU analysis also showed variation in the cell cycle distribution among the four subclones (Fig.2B), with a slight increase in the fraction of actively proliferating cells in U-251-FGA20gain (*P*-values: U-251MG = 0.057, U-251-4q12 = 0.004, and U-251N = 0.018). However, the proportion of actively proliferating cells does not correlate to the difference in growth rate, indicating that the total cell cycle time is longer for the U-251MG and the U-251-4q12 cells.

Clonogenic assays are commonly used to assess tumorigenicity of cell lines in vitro. We performed a comparative study of the clonogenic growth of the four subclones as well as their response to irradiation. In comparison with U-251N and U-251-FGA20gain, the U-251MG and U-251-4q12 colonies were much smaller and less dense (Fig.2C). One limitation with this clonogenic assay is that the definition of a colony is difficult if cells are migrating and showing dispersed growth. The U-251MG cells showed a high migratory pattern and the colonies were difficult to define. Consequently, these were not included in the calculation of PE and irradiation response. The PE was significant lower for U-251-4q12 (PE = 23.8 ± 4.47) compared to U-251N (PE = 42.5 ± 4.66, *P* = 0.0075) and U-251-FGA20gain (PE = 46.56 ± 7.70, *P* = 0.0115). Irradiation at 2 and 5 Gy show that U-251-4q12 cells are more sensitive than U-251N and U-251-FGA20gain (Fig.2D), however, the difference is only significant for U-251-4q12 compared to U-251-FGA20gain at 5 Gy (*P* = 0.0248). This indicates that the U-251 subclones with higher growth rate and more clustered growth pattern also show more clonogenic growth and a tendency to higher tolerance of ionizing radiation.

### Increased in vivo tumorigenicity after long-term in vitro culture

**Figure 4:**
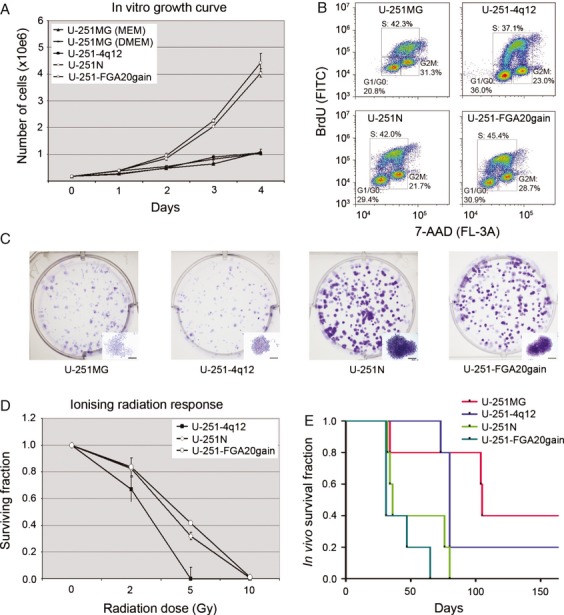
In vitro and in vivo growth U-251 clones. (A) Growth curves of U-251MG in MEM, U-251MG in DMEM, U-251-4q12, U-251N, and U251-FGA20gain. (B) BrdU incorporation shows various distributions of the S-phase cells. One representative figure is shown, while numbers are the mean from three replicate analyses. (C) Examples of variation in colony formation obtained by U-251MG (left), U-251-4q12 (middle left), U-251N (middle right), and U-251-FGA20gain (right). Single colonies are shown as insets with scale bars = 100 *μ*m. (D) Survival of clonogenic colonies after irradiation at 2, 5, and 10 Gy. U-251-4q12, U-251N, and U-251-FGA20gain. (E) Kaplan-Meier survival curves after intracranial implantation of the U-251 subclones in NOD/SCID mice.

In order to assess whether the variations in growth patterns observed in vitro were reflected in vivo, we implanted 50,000 cells intracranially in NOD/SCID mice to evaluate tumor take and survival time of the mice. The average survival of U-251-FGA20gain and U-251N was 41 and 52 days, respectively. In contrast, some of the U-251MG and U-251-4q12 mice survived more than 130 days, with average survival time of 111 days for U-251MG and 97 days for U-251-4q12 (*P*-values: U-251MG ≤0.0451 (U-251N) and <0.0134 (U-251-FGA20gain); U-251-4q12 = <0.0391 (U-251N) and <0.0050 (U-251-FGA20gain). In close agreement with the in vitro assays, the cells with a higher growth rate and higher clonogenicity in vitro also had a more aggressive growth in the mice (Fig.4E).

## Discussion

The aim of this study was to characterize the genetic, phenotypic, and functional differences of the original U-251 and various subclones of this cell line, including one that in numerous publications have been referred to as U-373 (named here U251-4q12). Similar variations may be found in any cancer cell line that have been extensively subcultured over many years, underlining the importance of strict cell culture routines and restricted time of subculturing according to cell bank guidelines.

aCGH is an important tool to identify genetic signatures common to different cancer types 19. The most prominent difference as analyzed by aCGH was the loss of a typical primary GBM profile for all subclones other than U-251MG. U-251MG has DNA copy number aberrations typical of a human GBM profile 19, with gains of chromosomes 3, 7, 15, and 17, and losses of chromosomes 10, 13, and 14. The long-term cultured subclones U-251-4q12, U-251N, and U-251-FGA20gain had lost these characteristics. These three subclones all carried a deletion of 18q11-23, which has been described as a cell culture-induced phenomenon in GBMs 17 and also in cell lines derived from other tumor types 20. The reason for this is not known. Interestingly, the U-251-4q12 cell line has an amplification of chromosome 4. This amplification was not detected in the original U-251MG cell line, and may indicate that U-251-4q12 is derived from a different cell within the original U-251MG cell line, although it cannot be ruled out the possibility that this is a culture-induced amplification phenomenon. In this context it should be recognized that amplification of 4q12 has been reported in a subset of GBMs 21,22. The amplicon on 4q12 contains a cluster of genes including PDGFR*α*, KIT and VEGFR2. Interestingly, Holtkamp and colleagues 22 reported a remarkable amplification pattern in U-373 cells with a 19-fold increase in the central portion of the amplicon. Amplification of 4q12 was also detected in SNB-19. This can potentially be explained by the fact that both of these cell lines are of the same origin as U-251 7.

GBMs frequently carry mutations in PTEN and TP53 as well as p16/p14ARF deletions 23. Mutations in these genes have also been reported for U-251 5,6. By aCGH we confirmed that the p16/p14ARF locus was homozygously deleted in all U-251 subclones.

The STR profiles among the four U-251 clones are very similar, with some LOH at some loci. The allele gain at FGA locus 20 in the subclone U-251-FGA20gain does not seem to affect the character of the cell line, with respect to its growth pattern, morphology, proliferation, clonogenicity, and radiation response. It also does not seem to change the genetic profile, when analyzed by aCGH. The U-251MG and U-251-4q12 cells clearly differ from the other two U-251 clones by all parameters measured in this study. Both U-251MG and U-251-4q12 cells are morphologically larger and grow flatter and more evenly distributed in the tissue culture flasks compared to the two clustered subclones. Furthermore, they proliferate more slowly and are less tumorigenic in vivo. However, both the STR analysis and the aCGH clearly show a common origin for all U-251 clones.

When choosing an in vitro model for cancer, the most important criteria are that the model reflects the tumor type it is supposed to represent and that the correlation between tumor model and tumor type is high. The genomic profiles of human GBMs cultured as monolayers are frequently deviant from the parental tumors and during prolonged culture genetic changes accumulate 24. Quite disturbing are data showing that cell lines appear more similar to each other, regardless of the tissue of origin, than the tumors that they are supposed to model, both with respect to their genetic profile and at the transcriptional level 18,25. Furthermore, in a comparative study to investigate the multidrug resistance transcriptome of various cancer cell types, no correlation was found between clinical samples and established cancer cell lines 25. Yet, one study concluded that commonly used breast cancer cell lines to a large extent reflect the features of cancer cells in vivo 26.

In this study, we found a clear variation in the aCGH profile of the long-term passaged U-251 subclones compared to aCGH profile of low-passaged U-251MG. During long-term subculture the cell lines seem to lose much of the typical GBM signature, and they gain a large number of local duplications and/or deletions that probably accumulates over time. This phenomenon may obscure comparative results obtained by testing drug effects in different laboratories.

Since several of the major cell deposits have distributed the cross-contaminated U-251, there is reason to believe that most articles referring to U-373 are in fact referring to the subclone U-251-4q12, in particular those submitted before the correct U-373 was made available at HPA medio 2010. In order to fight the problem of cross-contamination, several journals now require cell line verification before publication, yet there are continuously new articles published with experimental data from the U-251 and U-373 cell lines, in which it is not obvious if the authors have used the cross-contaminated U-251 or the correct one. Whether the cells have been authenticated before publication is thus not obvious to the readers. It is, therefore, highly recommended that cell line authenticity should be stated in the material and method section of all published articles along with the cell source.

In summary, cell lines represent simple model systems that have contributed extensively to elucidate various biological functions of cancer cells. In addition, the glioma cell line U-251 and other cell lines isolated by the group in Uppsala, Sweden, have provided important new insight into GBM biology. Yet, given the high number of articles published each year related to the U-251 cell line, which in fact may represent different subclones with different biological properties, a further specification of the subclone used is mandatory. Such a specification, which is provided in this study, is important in order to justify in-depth comparisons of results obtained between different laboratories.

## Conflict of Interest

None declared.
